# Lameness in Beef Cattle: UK Farmers' Perceptions, Knowledge, Barriers, and Approaches to Treatment and Control

**DOI:** 10.3389/fvets.2019.00094

**Published:** 2019-03-29

**Authors:** Jay Tunstall, Karin Mueller, Dai Grove White, Joanne W. H. Oultram, Helen Mary Higgins

**Affiliations:** ^1^Department of Epidemiology and Population Health, Institute of Infection and Global Health, University of Liverpool, Neston, United Kingdom; ^2^Department of Livestock Health and Welfare, Institute of Veterinary Science, University of Liverpool, Neston, United Kingdom

**Keywords:** lameness, beef, locomotion, perceptions, qualitative, welfare, culling, prevalence

## Abstract

Lameness in the beef industry has received little attention in the UK, despite the fact that it is a well-recognised problem in the dairy industry. The aims of this study were to (i) compare UK beef farmers' estimates of lameness prevalence to that of researchers, (ii) explore beef farmers' attitudes towards lameness, and (iii) help identify farmer reported barriers to lameness control and treatment. Beef farmers (11 finishing units and 10 suckler farms) were recruited from England and Wales. Farmers were asked to estimate their lameness prevalence, before a researcher conducted locomotion scoring using a five point scale, and a Bland Altman analysis performed. Face to face interviews were also conducted using a semi structured interview script aimed at capturing information such as current approaches and protocols as well as their views of lameness importance. Interviews were recorded and transcribed. An inductive thematic analysis was performed. All but two farmers underestimated lameness prevalence on their farms when compared to the researcher. Farmers initially underestimated lameness prevalence compared to the researchers estimates, with a mean underestimate of 7% (95% CI 5–9%). This is an important barrier to lameness detection and treatment. Thematic analysis identified four major themes: (1). Perception of lameness prevalence, (2). Technical knowledge and skills, (3). Perception of the impact of lameness, and (4). Barriers to the treatment and control of lameness. This study highlights that some approaches to lameness treatment are likely to be causing harm, despite being done with the intention to help the animal. There were four key areas of concern identified: recognition of lameness, treatment approaches, the training provided to farmers and confusion over transport and slaughter options available to farmers. This suggests an urgent need for future work to quantify and address the problem, and to provide evidence to justify the role of prevention and potentially start to break down barriers to control and treatment of lameness.

## Introduction

Lameness is well recognised to be a problem in UK dairy industry, as well as internationally (1, 2). It is considered to be one of the top cattle health and welfare challenges (3), and is considered to cause considerable pain and distress to cattle (4). In dairy herds, recent reports have estimated the within farm prevalence to be 31.6% with a notable amount of variation between farms (5). However, there is little known of the prevalence of lameness in beef cattle, particularly in the UK. A Norwegian study identified a lameness prevalence of 1.1% in suckler herds, although claw and limb disorders were identified in 29.6% of animals (6). A University of Nebraska review of records on US feedlots showed 2% of cattle were treated for lameness, and it accounted for 5% of animal deaths (7). However, this study required lame animals to be identified, treated and recorded, which risks underestimation if they are not identified, or if the lameness is not treated, or if it is not recorded. Furthermore, both of these studies were of cattle in different husbandry and management conditions to UK beef cattle. Some studies have sought to compare dairy farmer reported estimates of prevalence to that of researchers. These have shown that dairy farmers are typically underestimating lameness within their farms (8, 9). However, beef farmers estimates of lameness prevalence have not been studied. If farmers do not accurately assess lameness it is likely to be a fundamental barrier to tackling the problem.

Even if farmers can accurately estimate lameness prevalence within their herds, that alone does not necessarily equate to action being taken. There has been some attention given to farmer perceptions and motivations in a broader sense. A 2011 review explored New Zealand dairy farmer decision making, with a particular focus on veterinarians motivating dairy farmers (10). This review discusses how farmers may not act on advice, despite the promise that an action will improve a situation. The review considers how this lack of uptake of advice may be due to a number of factors including self-confidence, habit and desire to maintain simplicity, amongst other factors. Valeeva et al. studied motivation to improve dairy cattle mastitis on Dutch farms, and found that motivators could be categorised into three groups: those focused on penalties or premiums, those driven by a desire to have an efficient farm that meets regulations, and those motivated by simple economics (11). However, Hansson and Lagerkvist concluded that the most important factor within a study of Swedish dairy farmers' motivating values regarding dairy cattle mastitis was for a farmer to be happy that their dairy cows are “well-kept” (12).

Farmers' approach to dealing with the risk of a new issue, or a current issue getting worse could be important when considering lameness prevention, as shown by Garforth et al. They performed an interview study of UK pig and sheep farmers, considering risk management, and highlighted how farmers' actions following advice are strongly related to their attitudes towards risk, and how they were more likely to react to a current local situation rather than to prevent the silent spread of an unknown disease (13). The authors also discussed how farmers' perceptions of risk are different from the veterinary profession and from Defra. This study also identifies that farmers were willing to change habits, but require sufficient convincing to do so. This indicates that even if a specific lameness risk is known by farmers, a willingness to take risk can affect the uptake of any prevention strategies. It also highlights how that may lead to a difference in opinion between different areas of the industry.

Industry collaboration is likely to be important in preventing lameness, providing knowledge as well as treatment options and services. However, this may be difficult with differences of opinion, and may be made more difficult if the industry cannot provide these when required. Kaler and Green identified that UK sheep farmers' perceived that their veterinarians have insufficient knowledge in flock health planning and of the farmer's own circumstances to be of value for flock level planning (14). This contrasts with the study by Garforth et al., where veterinarians were considered as the most credible and relevant source of disease information, and may even be used to filter, fact check, or even summarise new information (13). However, when considering cattle lameness, a questionnaire study of Dutch dairy farmers identified that the feed advisor and the foot trimmer appear to have the most influence on the farmers' intentions to improve (15). These contrasting reports may represent either different stages of a changing picture of influential roles, or that there is variation between livestock sectors or between geographical areas. It is likely to be important that whoever a farmer is influenced by can provide adequate knowledge and support.

It has been demonstrated that farmers might find defensive reasons why they are unable to meet specific requirements. Naylor et al suggested that farmers may blame government organisations for failings in disease outbreak situations, or the uncontrollable nature of a disease (16). The authors reported specific differentiation by some participants between “good” and “bad” farmers, with bad farmers being responsible for problems within the industry. Farmers from the poultry and pig industries in particular were likely to stratify their industry, with “hobby” farmers being more likely to be perceived as “bad” farmers. A UK cattle and sheep study also identified that farmers blamed policy and regulations for previous disease outbreaks, justifying the lack of action they were taking, as well as considering some diseases to be only a problem for “bad” farmers (17). This blaming of organisations such as the government may have an impact in the likelihood of advice from these sources being accepted and utilised in the future (18).

In terms of cattle lameness perceptions, Bruijnis et al., the authors of the Dutch questionnaire study (15), also identified that 25% of the respondents did not perceive that cattle can experience pain. This may be due to the stoical nature of cattle masking the signs of pain, but may suggest that these farmers perceive that their cattle are well-kept even if lame, and as such there may be a reduced drive to resolve lameness.

There have been qualitative studies seeking to explore the perceptions of lameness amongst dairy farmers (19), and the motivators and barriers to its control, and while this existing literature does provide useful insight into farmer perceptions and barriers that are present, to the authors' knowledge, there have been no equivalent types of qualitative studies in the UK beef industry. There are clear differences in terms of management and husbandry between the dairy and the beef industries, therefore, it is not necessarily appropriate to directly extrapolate our current knowledge of lameness practices and perceptions within the dairy industry to the beef industry.

To help to fill this gap in the literature, the aims of this study were (i) to compare UK beef farmers' estimates of lameness prevalence to that of researchers, (ii) to explore their attitudes towards lameness and, (iii) to help identify farmer reported barriers to lameness control and treatment.

## Methods

This study was approved by the University of Liverpool Veterinary Research Ethics Committee (VREC 533). It is reported in accordance with the consolidated criteria for reporting qualitative research (COREQ) checklist, see Appendix 1 (20).

### Identification and Recruitment of Beef Farmers

A sample size typical for this type of research study was determined, allowing identification, and exploration of key opinions and insights. Guidance for this sample size came from Guest et al. (21), who discuss how with increasing sample size, the new themes and even the number of new codes decreases. Based on this guidance it was decided to initially recruit approximately 10 finishing unit farmers and 10 suckler herd farmers as these can be considered as two distinct important sectors within the beef industry. As data was accrued, it was continually assessed for saturation, and after 21 interviews the final assessment was made, where it was deemed that saturation had occurred. The inclusion criteria for the suckler herds were having suckler cows housed at the time of study (January–April 2018). The inclusion criteria for the finishing units were having finishing cattle housed at the time of study (June–October 2017), on their final ration, and due to be sent to slaughter directly from the farm. Farms having <60 suitable animals were excluded to minimise the impact of lameness prevalence estimates varying due to single animals. This could not be based on a sample size calculation due to the lack of pre-existing data. Convenience sampling and snowball sampling were employed. Twenty farms were recruited via the professional contacts of the researchers, including approaching 32 veterinary practices and 18 industry bodies. One hundred fifty farms were approached by JT, and were also asked to suggest other potential participants. One farm was recruited via this snowball sampling.

### Data Collection

Face to face interviews were conducted by JT at the farmer's address with the person responsible, or jointly responsible, for making management decisions on farm. A semi structured interview script was designed by the authors and piloted with two farmers (see Appendix 2). The pilot data is not included in the data set. The questions were a mixture of open and closed questions. The main topics covered were (i) current approaches to individual lame animals, (ii) herd lameness prevention plans, and (iii) understanding of the effect of lameness on farm. Farmers were asked about current and previous cases of lameness on their farms, including discussing how they identify and treat lame animals.

The interviewer ensured all questions were asked, using prompts where required, but farmers could choose not to provide an answer. The interviewer allowed flexible discussion, encouraging exploration of responses. Lesion pictures were available to confirm descriptions [taken from Archer et al. (1)] and drawings were encouraged when appropriate. The interviews were audio recorded by the researcher and transcribed verbatim with secretarial support.

Farmers were also asked how many lame animals they had (within the group in question – cows/finishing cattle). Following this they were then presented with the information in Table 1, and asked how many animals they had of each score. The scoring system was a five point modified scale combining that used by Sprecher et al. (22) and one promoted by the Agriculture and Horticulture Development Board (23). Any animal scoring two or above were classed as lame.

**Table 1:** Locomotion scoring system used.

**Score**	**Category**	**Description**
0	Normal	Even weight bearing and rhythm on all four feet. The back is level.
1	Imperfect locomotion	Uneven steps or shortened strides, but affected limb not identifiable. The back may show minimal arching while walking.
2	Impaired locomotion	Uneven weight bearing or shortened strides. Affected limb is identifiable (unless multiple limbs affected). The back may show arching while walking.
3	Severely impaired locomotion	Slower pace—Unable to keep up with the healthy herd. Affected limb easily identifiable (unless multiple limbs affected), but whole foot placed to floor. An arched back may be noted while standing and walking.
4	Severely impaired locomotion with non-weight bearing limb(s)	Slower pace—Unable to keep up with the healthy herd. Affected limb easily identifiable (unless multiple limbs affected). An arched back may be noted while standing and walking. One or more limb non-weight bearing or toe touching.

*It was based on two systems, one used by Sprecher et al. and one promoted by the Agriculture and the Horticulture Development Board (22, 23)*.

Farmers were either interviewed before locomotion scoring took place (20/21), or were absent for the locomotion scoring, and interviewed afterwards without knowledge of the results (1/21). The process of locomotion scoring varied slightly on farms depending upon facilities available, but typically cattle would be run through a purpose built handling system, where their official ear tag or management tag were recorded, and then cattle were locomotion scored on leaving the handling system. An alternative process involved releasing animals individually from a gated holding pen, and a management tag being read on release. In all cases, the cattle were individually identified and then scored on a hard surface, generally concrete. If the researcher needed a second opportunity to view an animal, the animal was either returned to the handling system or released again from a holding pen. Locomotion scoring was carried out on all farms by JT either on the same day as the interview (*n* = 20), or within 5 days (*n* = 1). In the case of the latter, the farmer reported no change in lameness rate between day of interview and day of researcher scoring. On some farms, it was not possible to locomotion score all eligible animals for logistical reasons. Therefore, a pragmatic decision was made based upon what could be achieved in one day using the facilities available. This did mean that on some farms, fewer animals were locomotion scored than the number required for recruitment onto the study. These farms remained within the study. Although farmers had some control over which animals/pens were chosen, it is the authors' belief that this choice was based on logistical or safety reasons, rather than an attempt to manipulate the outcome.

### Data Analysis

An inductive thematic analysis was performed on the interview transcripts as described by Braun and Clark (24) using NVivo qualitative data analysis software, (QSR international Pty Ltd. Version 10, 2012) by JT and HMH. Themes were refined following discussion, while ensuring that they were directed by the data. This included frequent reference to both the coded extracts and the transcripts to ensure that the themes represented the data.

Microsoft Excel (Microsoft Office Professional Plus 2013, version 15.0.5041.1000) was used to record and analyse the quantitative data. Bland Altman plots were used to compare farmer to researcher locomotion scoring estimates.

## Results

### Characteristics of Participants

Interviews lasted between 24 and 78 min. The study included 5 farms located in the North West of England, 3 in the West Midlands, 1 in the East midlands, and 12 across North Wales. All interviews included at least one of the main decision makers, but some included more than one partial decision maker for at least part of the interview. The main interviewee in 20/21 interviews was male. The exception was a joint interview with one male and two females, all responsible for management decisions on farm. The mean age of the main interviewee was 49 and ranged from 27 to 72 (one farmer declined to provide an age). Out of the 21 main interviewees, 15 (71%) had attended an agricultural college or university. The median total number of cattle on the farms was 285 at the time of interview, with a range of 100–800. This includes cattle ineligible for study (for example breeding bulls and young stock). The median number of eligible cattle on the farms was 120, with a range of 59–525. The mean number of cattle locomotion scored was 91 (Range 49–133). All eligible cattle were scored on 13 farms, 62–75% of eligible cattle were scored on three farms and 20–37% of eligible cattle were scored on 5 farms. Lameness prevalence as scored by the researcher ranged from 0 to 43%.

### Beef Farmer and Researcher Estimates of Lameness Prevalence

**Figure 1:**
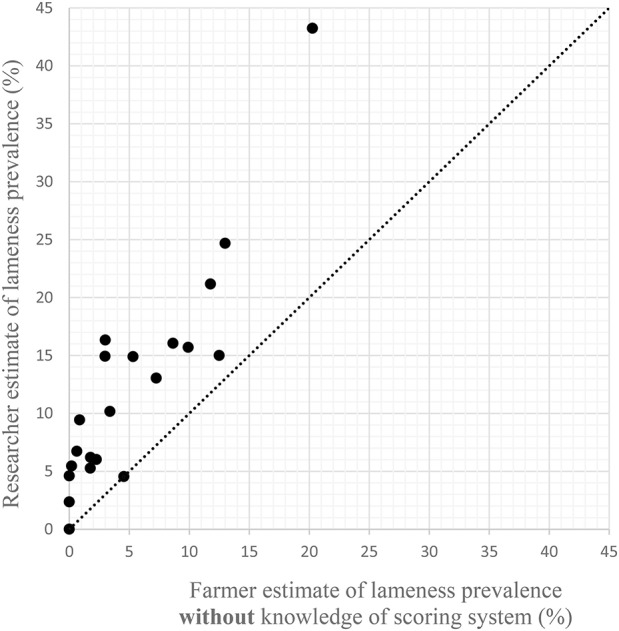
Scatter plot of researcher's estimates of lameness prevalence following locomotion scoring against farmers' estimates **without** knowledge of the scoring system to be used. The line shows equivalence.

**Figure 2:**
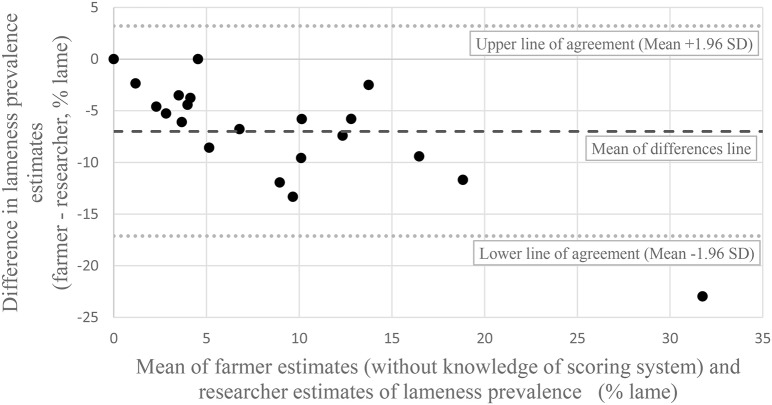
Bland Altman plot of farmer estimates of lameness prevalence **before** being shown the scoring system and the researcher estimates of lameness prevalence from locomotion scoring.

**Figure 3:**
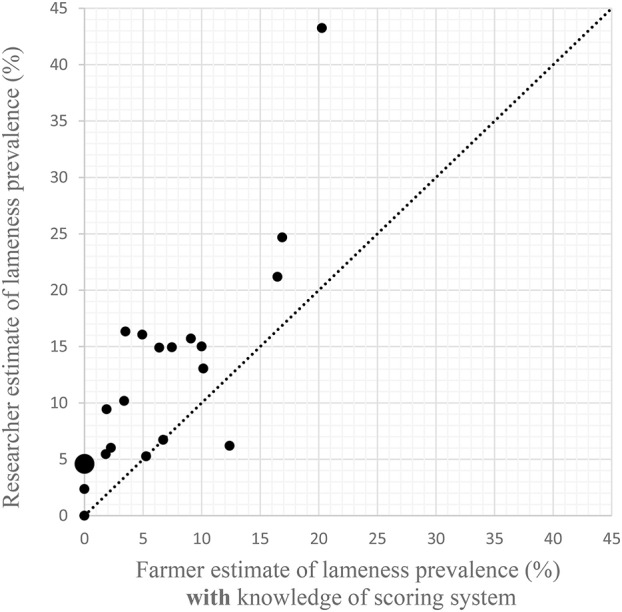
Scatter plot of researcher's estimates of lameness prevalence following locomotion scoring against farmers' estimates **after** being shown the locomotion scoring system. The line shows equivalence. Large data point represents the values of 2 researcher/farmer results with overlapping responses.

**Figure 4:**
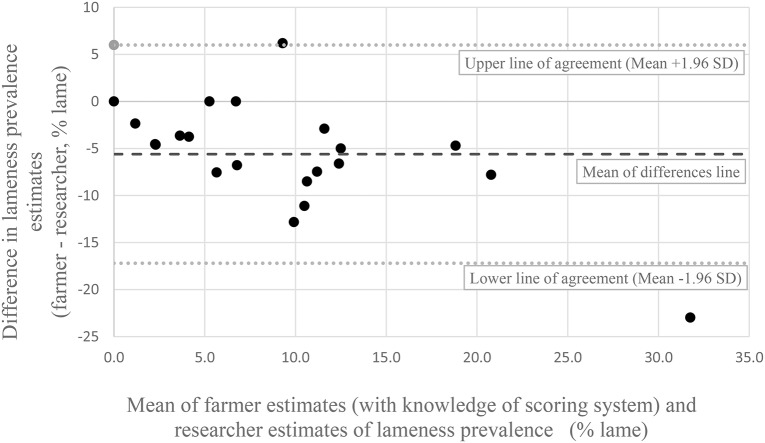
Bland Altman plot of farmer estimates of lameness prevalence **after** being shown the scoring system and the researcher estimates of lameness prevalence from locomotion scoring.

Without knowledge of the scoring system, all but two farmers estimated a lower prevalence of lameness than the researcher Figure 1). The remaining two farmers estimated the same prevalence as the researcher. The Bland Altman (25) plot (Figure 2) show that the mean difference between the farmer without knowledge of the scoring system and the researcher was −7% (95% CI −5 to −9%). The upper line of agreement was at 3% (95% CI −1 to 7%), and the lower line of agreement was at −17% (95% CI −13 to −21%). This represents a 20 percentage point difference in lameness estimate, and shows that farmers could be expected to be 3 percentage points higher in their estimate, or 17 percentage points lower than the researcher. With knowledge of the scoring system, three farmers estimated the same percentage as the researcher, and one farmer estimated a higher prevalence than the researcher (Figure 3). The remaining 17 farmers estimated a lower prevalence of lameness than the researcher. Figure 4 shows that the mean difference between the farmer with knowledge of the scoring system and the researcher was −6% (95% CI −3 to −8%). The upper line of agreement was at 6% (95% CI 1 to 11%), and the lower line of agreement was at −17% (95% CI −13 to −22%). This represents a 23 percentage point difference in lameness estimate, and shows that farmers could be expected to be 6 percentage points higher in their estimate, or 17 percentage points lower than the researcher. The differences between the farmer and the researcher of 20 and 23 percentage points would not be clinically acceptable.

**Figure 5:**
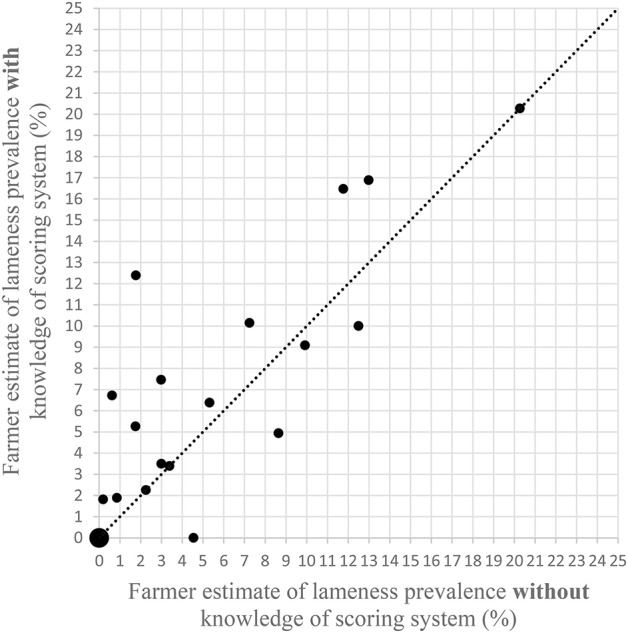
Scatter plot of farmers' estimates of lameness prevalence on their farm before and after being shown the locomotion scoring system in Table 1. The line shows equivalence. Large data point represents the values of 3 farmers with overlapping responses.

The change in farmer's estimate of lameness prevalence before and after being shown the locomotion scoring system was variable (Figure 5). Some farmers reduced their estimates (*n* = 4), some kept the same estimate (*n* = 6), however the majority (*n* = 11) increased their estimate. One farmer increased their estimate from <2% to over 12% after seeing the scoring system.

### Thematic Analysis

**Figure 6:**
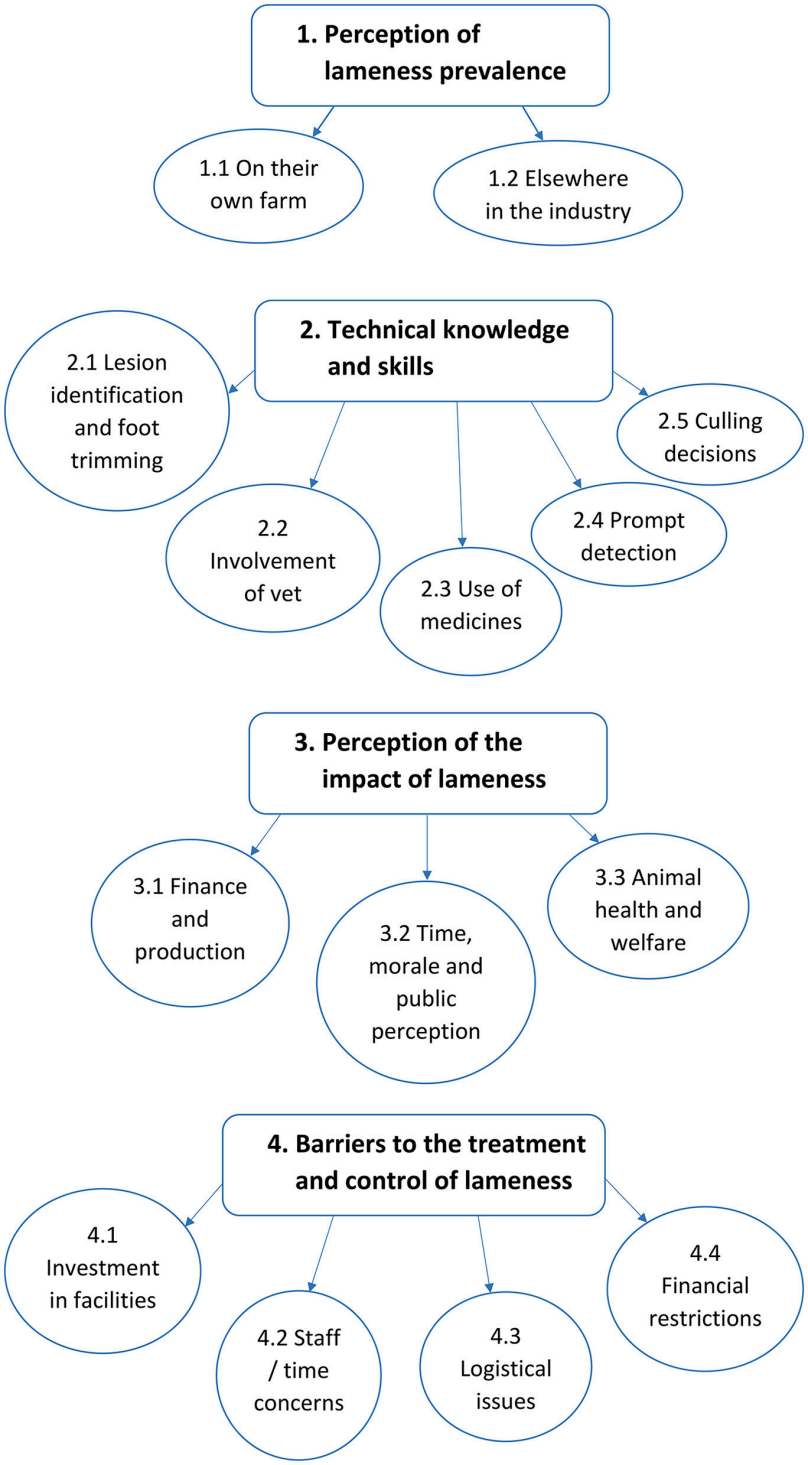
Flow diagram showing the four major themes, and their respective minor themes identified from the data following thematic analysis.

Four main themes were identified during analysis, with a number of sub themes: (1) farmers perception of lameness prevalence, (2) technical knowledge and skills, (3) farmers perception of the impact of lameness, and (4) Barriers to the treatment and control of lameness (Figure 6).

#### Theme 1: Farmer Perception of Lameness Prevalence

There are two sub themes, each described below. Record keeping, which may enable monitoring of lameness prevalence was variable. Some farmers reported keeping full records, although many felt that they knew in their heads who the chronically lame animals were. However, on investigation, records were generally only kept if drugs were administered. In some cases, these records were vague. In addition, some farmers reported only starting to keep records once they had a serious lameness problem at herd level.

##### Farmer perception of lameness prevalence on their own farm

One farmer acknowledged how they struggle to identify lame cows:

“*Yes, it's hard to see without having them walking, because they're housed inside, they don't walk much.”*

“*Anything slightly lame, on straw, doesn't always show as easily as something on concrete”*

In addition, one felt that it can become normal for them to be lame:

“*We're used to seeing her terrible, so you don't really… They are probably never not lame actually.”*

None of the farmers were using a formal lameness scoring system, and most reported that they look for lameness when feeding, bedding or scraping passageways, meaning animals are observed while on various surfaces, including deep straw bedding. Limping, hobbling, not fully weight bearing or being slow to get up/refusing to get up were the most common things looked for in identifying lame animals. This list also included not eating, observed swelling or redness and being able to “*just tell”*.

Farmers also frequently commented that what they considered to be lame may differ from what a researcher may consider lame. Furthermore, when looking at the scoring system, some farmers did use language indicating that they were either trying to second guess what the researcher might say was lame, or exaggerating the number of animals of each locomotion score:

“*I can't think of any [of that score], but put two for that.”*

In addition, farmers described some animals that they would not call lame, helping to identify where their threshold may be when asked if there are any lame cows:

“*There are one or two that aren't carrying their full weight, but they are not… [Farmer trailed off].”*

Hoof shape caused notable confusion amongst a small number of farmers, as some would call any with abnormal hoof shape lame (regardless of how they walk or bear weight), whereas others would use hoof shape to excuse lame animals (that were scored as lame by the researcher), and not call them lame:

“*Erm, not got any lame ones but a couple have, er, where the hooves have grown in a particular shape.”*

Others would excuse animals from a lame list for other reasons:

“*…But it might not have been lameness, it might have been a hip problem, maybe.”*

Some farmers progressed to speak of how they felt that the way an animal walked may not be affecting the animal:

“*It's not bothering them too much, but you can tell he's not moving as he should be.”*

“*There's one that's lame. There are a couple of others that need foot trimming or maybe are just a tad tender.”*

There was variation in the abilities of farmers to examine lame animals, as some could not lift feet at all with the facilities available, and some could only lift the back feet. Some said that they could lift feet, but did not feel that it was safe to do so.

##### Farmer perception of lameness prevalence elsewhere in the industry

Farmers were asked how they felt any lameness on their farm compared with other similar units. Most reported that they had little idea of what lameness was like on other similar units. Furthermore, many farmers appeared to have little access to other similar units:

“*I've no idea, I don't know what other beef units do.”*

#### Theme 2: Technical Knowledge and Skills

There are 5 sub themes within technical knowledge and skills. Notably, almost three quarters of the farmers had been to college or university. However, one felt it had not helped:

“*…For all the good it is…You can learn as much at home, to be honest with you.”*

##### Lesion identification and foot trimming

Many farmers described how they had or had not learned to trim cattle feet. Although some had learned in college or similar, a number were not confident and therefore not willing to trim feet. Others reported that they were self-taught using a variety of methods:

“*A bit self-trained I think. When we had the horses we used to do all our own farriery. So I do know a bit about things like that.”*

“*It was self-explanatory. A bit of common sense, you try to trim the feet like the feet should be – you know, square and flat and round and whatever.”*

Some farmers were using power tools to trim feet, or considering trying them. This included farmers reporting to have had no training in cattle foot trimming.

Farmer knowledge of lesion types was variable, but generally limited to a small number of lesion types:

“*So basically, I'm assuming anything that's not foul is digi [digital dermatitis].”*

Furthermore, terminology and communication of lesion types often required drawings, pictures or descriptions as names were not known or potentially confusing:

“*We do see blisters…like a soft putty bleeding lump.”*

Some practices were employed that indicated a need for further awareness of the underlying causes of lameness lesions, as well as the welfare of cattle. For example, when discussing how sole ulcers were dealt with by a farmer:

“*[I] burn them out with dehorning iron.”*

No farmers reported using routine, preventative foot trimming. However, some farmers did use an external foot trimmer, either expecting to book them a certain number of times a year, or just calling them as and when they felt they were required. However, some farmers reported difficulties in getting hold of a trimmer, either getting them within a suitable time frame, or at all:

“*He didn't even bother to turn up because he had plenty of better customers than one animal, that's the general feel.”*

“*He's got his own set timetable. He can only fit us in on cancellation.”*

##### Involvement of veterinary surgeon (Vet)

Farmers repeatedly reported that their vets have little involvement with lameness on their farms, and when they do call their vet, it is once the lameness is “*really bad”*.

Farmers mentioned cost, not knowing how their vets could help, or vets not being able to provide a “*magic injection”* as reasons for not involving the vet more. Furthermore, some farmers questioned whether their vets were able to provide suitable advice:

“*…They look more on the dairy side, well I don't think you can compare the dairy side and the beef side. So it's a job for them to… They would give us advice I think, but would it be the right advice because they look more on the dairy side?”*

Three quarters of farms had written herd health plans. All but one farmer said that they would not look at their plan if they had a problem on farm. The one farmer that said that they would, did not have lameness written within their plan. Some did not know if their plan had lameness mentioned within it. Most farms spoke negatively of the written herd health plan:

“*…It's a hoop we have to jump through. I don't see it as being particularly helpful to us, to be honest. It's just something we have to do.”*

##### Use of medicines

Farmers showed varying opinions towards antibiotic treatments, with some treating all lame animals identified, without reaching a diagnosis:

“*We injected all of them with Tylan [tylosin] at one stage when it first began…we put Linco-Spectin [lincomycin and spectinomycin powder] on and gave them a course of Tylan.”*

Whereas others would avoid antibiotic treatment for differing reasons:

“*We wouldn't jab it to start with because of the withdrawal period really.”*

Or one farmer's opinion after reporting that they were advised to use ceftiofur.

“*I'm not over keen, being a third-generation drug, and the abattoirs don't really want us to…I tend to shy away from those.”*

One farmer reported that they never use any drugs for lameness reasons. Some reported that the severity of lameness, rather than the diagnosis, would determine whether they use antibiotics, or would change the type of antibiotic used. Others stated that although they administer treatments for lameness, they never lift the feet of lame animals.

An off license lincomycin, spectinomycin combined powder treatment was mentioned as a treatment used by a number of farmers. Two had previously used it as a herd or group treatment in a footbath, and others had used it to treat individual cases. Some farmers appeared to discuss topical antibiotic treatments as if they were not an antibiotic:

“*We'll put the Terramycin [oxytetracycline] spray and a bit of Linco [lincomycin, spectinomycin combined powder] on it, bandage it up, and [depending] on how severe it is whether we give them antibiotics or not.”*

Some farmers implied a feeling of “better” antibiotics:

“*We've sort of ramped up the antibiotic armoury, going from a standard long acting penicillin through to Naxcel [ceftiofur].”*

Anti-inflammatory drugs were rarely given as part of lameness treatments, with only 4 farmers reporting that they might use anti-inflammatory drugs to treat some cases of lameness. Farmers reported not using anti-inflammatory drugs even in cases where pain was acknowledged to be involved in lameness.

Lameness vaccines were mentioned as something they would like to have available by a small number of farmers, linking with their knowledge of a vaccine being available for use in sheep. However, no link was made between the multitude of lameness lesions that might be found in cattle, and whether the causes of lameness on their farm was infectious.

##### Prompt detection

Some farmers reported that they do not always treat at first:

“*If it's a little bit [lame] you might leave it because it might have just sprained its leg. You'd leave it a bit before you'd do anything to it and then you'd get it in because it might have a stone in it or something like that.”*

Some felt that lameness will just get better irrelevant of treatment:

“*As I say, I'm not proud of saying it, but most of the time they burn themselves out.”*

##### Culling decisions

Most farmers acknowledged that they have had to cull, or prematurely slaughter animals due to lameness. Others had not, and felt that they keep lame cows that do not get in calf:

“*We do give them lots of chances before we actually sell them”*

“*But you see we're soft and we give everything a second chance.”*

Similarly, a finishing unit farmer acknowledged that one animal that was not culled was later regretted as it became more severely lame and could not travel, as well as being given treatment and being under withdrawal periods:

“*In hindsight, I wish he'd have gone, without injecting him sometimes you think it's better for him to go.”*

Conflicting experiences were noted with regard to what to do with lame animals that farmers wished to cull:

“*Maybe some people don't know what to do with a lame cow…you can send a lame cow [to the abattoir], can't you… [You're] better off getting rid of a lame cow than just having it hold its leg… Maybe some people need educating about what to do with lame cows, don't they?”*

In contrast, another farmer, when asked what was stopping him culling the lame animals reported:

“*We can't get them into the slaughter houses…If they'd let us go direct to the slaughter houses, them animals would be in less pain and out of the way quicker.”*

Another farmer acknowledged this as a “*minefield”*, and complained that the legislation was a “*grey area”*.

#### Theme 3: Farmers' Perception of the Impact of Lameness

There are three sub themes within this theme. Importantly, farmers held varying views on how they felt lameness impacted on their cattle, and their farm in general. Some felt that it was not a priority for them:

“*I don't think a lot of suckler farms are that worried with lameness. I think it's more of an issue with dairy farms”*

##### Financial and production impact

Some farmers did perceive that lameness negatively affects fertility. Some also noted that lame cows can produce less milk, having an impact on calf growth rates. However, for one farmer the costs were limited:

“*As long as it's still breeding a calf, it doesn't have a cost. The cost is, if it isn't in calf.”*

Furthermore, many suckler and finishing unit farmers acknowledged that lame animals can lose weight, or at least have decreased growth rates. Some finishing farmers appreciated the effects of this:

“*They get pushed through the finishing system…but obviously months behind.”*

However, others felt that the effect had to be severe to be worth intervening:

“*So as long as those feet aren't that severe and that it stops them eating and putting weight on, then we just leave them……………it's economics, okay?”*

A second farmer went further:

“*….it never knocks them off their grub.”*

A small number of farmers mentioned that animals had died, or they felt they had nearly died, due to lameness:

“*You could lose the beast if you let it get bad enough.”*

Some farmers discussed a concern regarding the contagious risk of lameness. They felt that the potential for spread could multiply the impact on their farm. However, it was not acknowledged that this may be similar for non-contagious lameness causes, where it is equally likely that all animals are exposed to the same risk factors as an animal that has become lame.

Many farmers did not appear to appreciate the indirect costs that may be attributed to lameness, however, some farmers did:

“*You're taking up space in sheds with animals that should have gone but are still on the farm”*

A lot of farmers interviewed did state that they were aware that lameness did cost them financially, although none were confident of how much lameness was costing:

“*No, I wouldn't have a clue. I'm sure it's quite considerable if you were to put pen to paper and add it up.”*

This lack of awareness of specific costs was repeated in numerous areas, as indicated by one farmer who felt that he did not want to spend money on preventative measures because:

“*The cost of prevention can be more than cure, at the minute.”*

However, when asked about what the actual costs were, an answer could not be given.

Some farmers did highlight that it can be difficult to appreciate the impacts of lameness, using lame animals on dairy farms as a comparison:

“*Erm, well performance because they don't milk the same, we should be the same with beef because they don't perform.”*

And a second farmer described it as:

“*… A hidden cost, because you don't physically see the money going out of your pockets… That's what I mean about farmers… You don't physically see the money, then you don't really know.”*

##### Impact on time, morale and public perception

The impact of lameness on a farmer's time was repeatedly mentioned. For some farmers it was a negative impactor on their time when discussing herd level prevention and individual treatment:

"It is a nightmare really, it wasn't a problem, and then suddenly became like, we're trimming feet all the time…”

The effects on farmer and staff were variable. For example:

“*Well, I don't see how that's going to affect the morale of the staff, I don't see where that should come into it.”*

This contrasts with the experiences of other farmers:

“*The constant battle we're fighting and not winning is mentally… what's the word… deflating.”*

Some farmers felt that having lame animals in a visible location, for example near a public road, might affect the public view of farming, but this was often felt to be more of an issue for both the dairy and the sheep industries than the beef industry.

##### Animal health and welfare

Importantly, some farmers spoke of how they did not feel that what they considered to be lame was significant enough to take action, both on a herd level, and an individual animal level:

“*If she's slightly hobbling, we tend to leave them… if you can tell she's in distress, we will have a look at them.”*

When asked generally about the down sides of lameness, some farmers mentioned some individual cow related factors:

“*Pain, you don't want it in pain, in distress, or anything like that.”*

And some added how having to get the animal out and treat it may cause additional stress. However, many farmers did not mention pain or welfare of the cow until asked more specifically about whether lameness has a welfare or pain component. Some farmers even compared it to how they would be in pain if they were lame. However, for some it was not clear cut:

“*Depending on the severity, yes”*

However, one added to this:

“*Yes, when they get to a point, score three or four…From a welfare point of view yes, you need to sort it out, but it's not your doing. They just go lame don't they?”*

#### Theme 4: Barriers to the Treatment and Control of Lameness

There were a large number of farmer perceived barriers to lameness control and treatment that were identified during interview. Although some of these barriers have been revealed in the previous three themes, others were identified which farmers perceived were reducing their ability, or likelihood of treating or controlling lameness.

##### Investment in facilities

Farmers mentioned a number of barriers which stopped them investing in their farm. Some rented all or part of their farm, and so wanted investment from the landlord to improve the facilities. Others felt they could not handle or footbath animals when they were outside as they did not have suitable facilities to do so. Some farmers presented general concerns regarding hesitation in making expensive investments in their farm. When speaking about their own handling facilities, one farmer highlighted how without further investment, climatic conditions may stop treatment being performed:

“*…it needs to be inside, then the weather conditions don't alter it then, do they?”*

One farmer summed up their opinion on investment within their farm by stating:

“*The job doesn't pay”*

##### Staff/time concerns

There were a number of time/staff issues which were identified as barriers. Some farmers perceived that during some periods they did not have time to do some things that might help prevent lameness (foot bathing in this case):

“*We stopped because of the amount of time it was taking…We got towards spring time and there were other jobs that wanted doing.”*

Whereas, others felt some jobs required more staff:

“*I don't think a footbath is practical here…because you're on your own.”*

##### Logistical issues

A number of logistical barriers were discussed by farmers. Some farmers interviewed were trying to increase their herd size, and as such did not want to cull any animals. This means that non-resolving lame animals would remain in the herd. Others could not cull a cow they had intended to because she was pregnant. As discussed in Theme 2, some farmers felt there was a grey area around transport and slaughter of lame animals, which acted as a barrier to culling.

Lack of slurry pit or waste (mainly faeces) storage was a barrier to more frequent cleaning out for some farmers, and the availability of certain types of bedding was affecting the choice made.

As discussed in Theme 2, concerns regarding withdrawal periods were a barrier to treating animals, and in particular the unknown duration of time an animal has left on the farm made it difficult for some farmers to decide whether to treat or not. Withdrawal periods were also a barrier to culling, as one farmer discussed following a long withdrawal period product applied on arrival to the farm:

“*…and if they injure themselves in the first week of coming here, we have to nurse them along until we can kill them.”*

Safety was discussed as a barrier for some farmers, safety of both themselves and their cattle, preventing them from examining and dealing with some animals. In addition, if a cow is heavily pregnant, or if the temperament may make it difficult to get the animal into the handling facilities, they may not do so:

“*I'm not going to get it into the crush if it's an idiot am I?”*

Farmers' identification and perception of lameness was identified as a specific barrier to the control and treatment of lameness. As discussed in Theme 3, some beef farmers see lameness as a problem for dairy farmers to worry about. In addition to the difficulty in identifying animals in straw bedding, or while inside housing, some farmers discussed how their finishing cattle will go to slaughter anyway, so they did not worry too much about some lameness, especially if animals still grow above a minimum threshold. Others felt that as some animals are permanently lame, they stop noticing them, whereas others simply do not consider lameness to be a problem with beef herds. Another farmer discussed how it was easier and quicker to spot performance deficits with dairy cattle, compared to beef cattle:

“*My* [dairy farming] *neighbour says if something is doing the cows no good, the milk is down. You don't see that with sucklers until something like six weeks down the line.”*

##### Financial restrictions

Financial barriers were also discussed. Variability in prices, for example straw, was used to explain why some farmers felt they were not doing what they would ideally or normally be doing. The cost of various potential treatments being perceived to be too high by some farmers, although little was known of the financial benefit of using the treatment discussed. Cash flow was also considered a barrier by some farmers, who may have felt an approach was worthwhile, but felt they could not go ahead with it. Some farmers also said that they are waiting for a grant to become available to assist in investment in new facilities. However, if no grant becomes available, or if lameness cases develop in the meantime, this dependence on potential grants will have been a barrier.

## Discussion

The aim of this paper is not to estimate the prevalence of lameness in beef cattle, rather to compare farmer's estimates with that of a researcher. The small sample size and the snowball sampling strategy, along with the combination of sucklers and finishers, make the prevalence of lameness identified potentially unsuitable for extrapolation to a wider population. However, it does highlight the variation that exists, which compares with the dairy industry, where there is also a large amount of variation between farms (5). It should be borne in mind that this study locomotion scored cattle during housing, from June to October 2017 for finishing units, and January to April 2018 for suckler herds, and as such may not take into account any seasonality effects on lameness prevalence. However, this should have little effect on the differences between prevalence estimates.

The difference between the upper and lower lines of agreement in both Bland Altman plots could be considered clinically important, with a difference of 20 percentage points for the farmers' initial estimate of lameness and the researchers estimate, and 23 percentage points when the farmer had knowledge of the scoring system. This means that we cannot use farmers estimate as an alternative for researcher estimates. This correlates with similar studies in the dairy industry (8). The fact that the locomotion scoring method has not been tested for intra or inter-observer reliability is a potential limitation of this study. Additionally, the researcher's awareness of the farmers' estimates prior to locomotion scoring can be considered a potential bias. However, the fact that the same researcher locomotion scored all cattle is a strength. In addition, the researcher was experienced in locomotion scoring, and had used this scoring system before.

The variation in age (and so likely time since education), as well as the different institutions and levels of courses attended, may mean that there are differences in previous teaching and exposure to locomotion scoring and lameness detection. For some, any exposure while in education may have been some years ago. This may reflect the differences in variation between different farmers and the researcher, with some farmers estimating the same as the researcher estimate, and some estimating notably less. However, we do not have data regarding any training since formal education.

Comparing Figures 1–3 combined suggests that presenting the information in Table 1 to farmers is not sufficient to enable them to assess the lameness in their herd. Combining this with the difficulties expressed by farmers in terms of identification of lame animals suggests that training and practice is required in order to enable farmers to improve the prompt detection of lame animals. Although some dairy cattle studies have suggested that training may provide limited improvements in intra- and inter-observer agreement when locomotion scoring (26, 27), inter-observer reliability has been shown to increase with increased time/scoring sessions (28). Also, experienced scorers have been shown to perform better than less experiences scorers when using video footage of cattle (29). This suggests that farmers can be assisted to improve their reliability in scoring. A 2014 review of locomotion scoring dairy cattle showed that although intra- and inter-observer reliability was variable for scales with over two levels, when the scales were considered at a lame/not lame level, all scoring systems exceeded the acceptance threshold (30), meaning that a binary locomotion scoring system may be best suited to on farm situations. This would be suitable where the next step from both a welfare and a production point of view would be further examination of any lame animals.

Although three quarters of farmers had been to college or university, Theme 2 suggests areas of weakness in both lameness knowledge and skills of beef farmers. Lesion identification, aetiology knowledge, and farmer description of foot trimming technique indicate an urgent need for further training to improve both the treatment and prevention that farmers can deliver for themselves. Crucially, some trimming techniques employed carried a significant risk of making a problem worse.

External support is not regularly being utilised, which is likely to be leading to suboptimal management of lameness, and reduced success rates. In particular, less veterinary time on farm, when compared to the dairy industry, may lead to less opportunity to ask questions and gain general information which a farmer may feel does not warrant a visit in its own right, but may be important in developing long term prevention and treatment strategies. This is marked when considering drug use, especially the lack of anti-inflammatory medication.

Herd health plans are generally written by both a farmer and their vet, and are required or at least recommended for assurance or certification schemes. They are often required to be reviewed and updated on an annual basis. The fact that herd health plans were not being used for lameness planning may not be surprising, as a Defra survey of farmers in all livestock groups, with over half of the respondents having a beef enterprise, showed that approximately half of the respondents claimed that health plans were effectively unimportant (31). It also compares with a study of dairy farmers where, despite overall mixed views, many felt that the main benefits to having a herd health plan were to meet external requirements, and that the plan was not in use (32). The fact that the farmers were not using their health plans, or did not have lameness covered within it suggests that this may be a missed opportunity. Ignoring the compulsory requirement for many farmers to have a plan, the process of reviewing and updating the plan provides an opportunity for the farmer to discuss lameness, as well as other performance and welfare parameters, with their veterinary advisor, and may enable appreciation of a problem, and discussion of improved solutions.

The approach to chronically lame animals was of particular importance: these animals can be expected to be in pain, yet potentially become trapped in a cycle of either being treated but not fully resolving, or not treated and not resolving, and therefore remaining lame. The variation in farmers' views suggests various experiences and that the information available is not clear, which is highlighted by one farmer calling it a grey area. Not being allowed to transport lame animals represents a barrier to culling these animals. The reported variation in whether an abattoir will accept lame animals leads to confusion and frustration.

There is a clear difference between farmers in their perception of the impact of lameness. For those who do not consider it to have a significant impact, there is less incentive to prevent it, or treat it as a priority. Furthermore, if farmers do not appreciate the full impact that less severe lameness can have both on productivity, and the welfare of the animal, some cases may be ignored. There may be some comparison with the study by Bruijnis et al. (15), and some farmers may not be perceiving that cattle can feel pain, or perhaps some don't perceive lameness as a painful condition. Although there is evidence detailing the impact of lameness in dairy cattle which can be provided to dairy farmers, to the authors knowledge there is no such data available for beef cattle.

The barriers identified are generally ones that can be overcome. If evidence can be produced, this could be considered the first step in breaking down barriers, and if the impact of lameness can be appreciated by farmers, there is potential for its order in a farmer's priority list to be elevated.

In terms of future work, establishing reliable and representative estimates for farm level prevalence of lameness within the UK will be important to quantify the scale of the situation, and research to provide evidence regarding the impact of lameness within beef cattle will be essential to give farmers and those advising them the confidence to invest in the prevention and control of lameness. In addition, identifying lameness detection methods that are suitable for routine use on beef farms will be of great value. However, this will need to be combined with a greater understanding of the complex interactions which lead to human behaviour change, and a full understanding of beef farmers' priorities. Therefore, further studies to understand both the barriers and pathways to change that exist for beef farmers would increase the potential for success.

Farm facilities represent a notable barrier to appropriate treatment. Farmers reporting that it is dangerous to examine lame animals using their facilities, or that their animals are likely to hurt themselves, means that significant investment incorporating foot examination facilities is required to ensure the safety of farmers and their cattle.

Farmer impressions that veterinary knowledge is mainly of the dairy industry highlights a barrier to requesting advice or assistance. A relationship needs to be established where beef farmers feel that they can trust the quality of the service of their vet, and the value that can be added by appropriate guidance and assistance.

One hundred and fifty farmers were approached by the authors during the recruitment process. The recruitment for this study may have led to a possible non-response bias. A small number of farmers (*n* < 5) who declined to participate suggested that lameness was not an issue for them, so it was less worthwhile participating. It is possible that farmers may not have wanted the researchers on farm if they had a substantial lameness issue.

## Conclusions

This research identified four key areas of concern. The first was the recognition of lame animals, including both ability and opportunity. The second was treatments, in that some treatments were likely to be directly harming animals, and some farmers were not promptly treating lame animals, both leading to a concern for the health and welfare of these cattle. Thirdly, the practical training provided to farmers was a concern. There was evidence that some farmers did not recognise a number of common lesion types and similarly did not know how to treat them. Finally, the study suggests that some farmers are confused over transportation and slaughter options for their cattle. This suggests an urgent need for future work to identify and address the scale of these concern, and to provide evidence to justify the role of prevention, and thus helping to break down some of the barriers to lameness control and treatment in beef cattle.

## Author Contributions

JT recruited participants, designed the interview script, conducted the field study, analysed the data, and wrote the manuscript. HH conceived the original study, assisted in designing the interview script, analysis of the data, and critical appraisal of the manuscript. KM conceived the original study, is PI on the grant, assisted in designing the interview script, and critically appraised the manuscript. DG and JO appraised both the original study design and the final manuscript. All authors have approved the final version of the paper.

### Conflict of Interest Statement

Farmers enrolled were paid a small financial sum which was determined to equate to the minimum time for one person required to partake in the interview, and facilitate locomotion scoring of cattle, as required for this research. The authors declare that the research was conducted in the absence of any commercial or financial relationships that could be construed as a potential conflict of interest.
