# Supplementary material for: Lameness in Beef Cattle: UK Farmers' Perceptions, Knowledge, Barriers, and Approaches to Treatment and Control
Source: Front Vet Sci. 2019 Mar 29;6:94. doi: 10.3389/fvets.2019.00094 (PMC6449762; doi:10.3389/fvets.2019.00094)
Supplement: Supplementary file 1 [file Data_Sheet_1.docx]

Appendix 1: Consolidated criteria for reporting qualitative studies (COREQ) table reporting level of adherence to guidelines.

| **Domain 1: Research team and reflexivity** | |  | |
| --- | --- | --- | --- |
| *Personal Characteristics* | | |  |
| Interviewer/facilitator | Jay Tunstall conducted all interviews | | |
| Credentials | BSc BVetMed MRCVS | | |
| Occupation | PhD Student | | |
| Gender | Male | | |
| Experience and training | Practice interviews, previous veterinary experience with farmers | | |
| *Relationship with participants* |  | | |
| Relationship established | Farmers were approached prior to the interview to discuss requirements of the study and arrange a suitable time and date | | |
| Participant knowledge of the interviewer | Participants were aware that the interviewer was a veterinary surgeon, researching the topic of lameness in beef cattle | | |
| Interviewer characteristics | The interviewer was conducting wider studies on the topic of lameness in beef cattle | | |
|  |  | | |
| **Domain 2: Study design** |  | | |
| *Theoretical framework* |  | | |
| Methodological orientation and theory | Inductive thematic analysis | | |
| *Participant selection* |  | | |
| Sampling | Convenience and snowball sampling were employed | | |
| Method of approach | Farmers were approached face to face, with further telephone calls to arrange a suitable time and date | | |
| Sample size | 21 farmers were studied | | |
| Non-participation | 150 farms were directly approached by the interviewer, as well as an unknown number by industry bodies. The main reasons given for non-participation were not wanting to give the time, not wanting to handle the animals due to the risk of stress or not being able to arrange a convenient time. A small number reported not feeling lameness was a significant problem, so it was less worthwhile participating | | |
| *Setting* |  | | |
| Setting of data collection | Interviews were carried out at the farm of the participant, at a location to suit them | | |
| Presence of non-participants | Some participants chose locations with other family / staff members present. Some of these participated, some were simply present in the background. | | |
| Description of sample | Demographic data is presented within the text | | |
| *Data collection* |  | | |
| Interview guide | The interview schedule is attached as appendix 2. It was piloted successfully with two farmers, and their data was not included | | |
| Repeat interviews | Repeat interviews were not carried out | | |
| Audio / visual recording | All interviews were audio recorded and transcribed verbatim | | |
| Field notes | Field notes were made during the interviews and locomotion scoring | | |
| Duration | Interviews lasted between 24 and 78 minutes | | |
| Data saturation | Data saturation was achieved in the main areas of interest | | |
| Transcripts returned | Transcripts were not returned to participants | | |
|  |  | | |
| **Domain 3: Analysis and findings** |  | | |
| *Data analysis* |  | | |
| Number of data coders | The data was initially coded by one researcher, but then refined by two researchers | | |
| Description of coding tree | The basic coding tree is displayed in Figure 4 | | |
| Derivation of themes | Themes were extracted from the data, without prior identification | | |
| Software | NVivo qualitative data analysis software, (QSR international Pty Ltd. Version 10, 2012) | | |
| Participant checking | Participants haven’t had the opportunity to feedback on the findings | | |
| *Reporting* |  | | |
| Quotations presented | Quotations have illustrated many of the findings | | |
| Data and findings consistent | The data is represented by the findings | | |
| Clarity of major themes | The major themes, as displayed in Figure 1, are presented in the findings | | |
| Clarity of minor themes | The minor themes, as displayed in Figure 1, are presented in the findings | | |
