# Supplementary material for: Lameness in Beef Cattle: UK Farmers' Perceptions, Knowledge, Barriers, and Approaches to Treatment and Control
Source: Front Vet Sci. 2019 Mar 29;6:94. doi: 10.3389/fvets.2019.00094 (PMC6449762; doi:10.3389/fvets.2019.00094)
Supplement: Supplementary file 2 [file Data_Sheet_2.doc]

Appendix 2: Interview Schedule used for all interviews

**Lameness in beef cattle: Establishing a knowledge base**

**In depth interviews**

Thank you for agreeing to take part in this study, I’m just going to remind you that you can ‘pass’ on any questions you would prefer not to answer, and also that your answers will be anonymous. The first few questions are just background information, so I can understand a little more about your farm. Please remember, there are no wrong answers. If I ask you ‘is there anything else you can think of?’, it isn’t because I think you’ve missed anything, it’s just because I want to make sure I’ve got all your opinions. You may feel some questions overlap. This allows you to express all opinions you may have.

| Total number of cattle on farm: |  | Are you the sole person responsible for herd management decisions?  Full / part time (%) | | | |  | | | |
| --- | --- | --- | --- | --- | --- | --- | --- | --- | --- |
| Management groups and other species on farm: |  | Are you the sole person responsible for day to day decision making?  Full / part time (%) | | | |  | | | |
| Farm type  Rented, owned, mix other enterprise |  | Do you have any formal training, qualifications or experience, either in farming or elsewhere? | | | |  | | | |
| Is beef farming your main source of income? |  |  |  |  |  |  |  |  |  |
| How long have you been farming beef cattle? |  | Gender: | | | |  | | | |
| No of regular workers / staff / family helpers:  Full / part time (%) |  | Age: | <20 | 20-30 | 30-40 | | 40-50 | 50-60 | 60+ |
| Location (county): |  |  | Prefer not to answer | | | | | | |

I’m now going to ask you some questions aimed at your finishing cattle / suckler cows, so please relate your answers to just that group of animals.

1. Please could you give me your current top three cattle health concerns?
2. Please can you tell me your most common reasons to cull animals (prematurely if finishing) in the last 12 m?
3. How many of each
4. (If lameness not A to Q1) You said lameness wasn’t in your top three concerns, is it something that concerns you?
5. Why / why not?
6. Already have control measures?
7. (If lameness not A to Q2) You didn’t mention lameness as a common reason to cull, have you had to cull any animals due to lameness in the last 2 years?
8. Please can you tell me about how you are dealing with your lameness issues?

OR

Please can you tell me about how you deal with any lameness issues, for example if you came across an issue tomorrow?

Tell me about a case that you were involved in…

1. How **ID**, knowledge / **training** of a formal scoring system
2. What looking for – **back**, **weight bearing**, head, feeding, any **records** kept
3. How examine – **crush** / race / lying
4. How treat – **trimming methods**, drugs, records, isolation
5. **Who** treat (internal / external)
6. When, **how long** following ID
7. Any herd level **controls**
8. Confirm ‘lesions’ / show 2 or 3 pictures
9. What involvement, if any, does your **vet** have
10. Any control measures already in place
11. Have you got any lame animals on farm today?

Y / N / unsure

1. (If yes to Q6 or 8) Tell me about any lame animals you have today, within the finishing cattle / suckler cows.
2. How many
3. Are they all of a similar **severity** – scale
4. **How long** have they been lame for
5. **Diagnosis** (inc. no of each) - pictures
6. (If no to Q6) Have you had any in last 1m (If no ask about 6m if no, ask about 12m? (If yes revert back to Q7 relating to this answer, if no, Q9)
7. Please could you tell me if this lameness / lack of lameness has changed over the last 12m (24m if no to Q6 & 8)?
8. Amount
9. Type / causes
10. I will be using a scoring system to grade how your animals walk. This involves watching each animal walking past me and giving them a score 0 – 4. I’m going to describe each score, and ask you how many animals you have of each score once I’ve described them all.
11. (Refer to Q5) You told me earlier on…. Is there anything you would like to be able to do or change to tackle your lameness situation / a lameness situation that may occur, but you aren’t currently doing / able to do?
12. Can you think of anything else
13. Please tell me what is preventing you from resolving an issue the way you mentioned you would like to? (refer to specific points)
14. Is there is anything else which you know of that may help, or others think may help to deal with lameness, inc. vets, advisors, farmers?
15. How do you feel lameness / lack of lameness on your farm compares with other farms?
16. Other finishing / suckler farms
17. Amount
18. Actions / controls
19. (If no lameness issue: Some farmers do have a lameness concern..) Is there anything which may assist, motivate or facilitate you (them) to deal with lameness?
20. **Vet** – proactive / enthusiastic
21. **Industry** involvement / advice
22. Do you have a HHP, and if so, does it include lameness?
23. What does it say about lameness?
24. Do you refer to it?
25. Do you use it to help you make decisions?
26. What would you say the downsides of lameness are, please mention as many as you can think of?
27. Are there any others you can think of?
28. Financial – treatments, culls, growth
29. Morale
30. Welfare / suffering / pain
31. Public perception
32. What would you say the benefits of having low levels of lameness are, please mention as many as you can think of?
33. Are there any others that you can think of?
34. Financial – treatments, culls, growth
35. Morale
36. Welfare / suffering / pain

Thank you very much, your time and help is really appreciated, and obviously your opinion is essential to this.

1. Are there any other comments you would like to add on this topic, or anything that we’ve discussed?

Get copy of foot trimming / scoring.
